# A novel peptide to enhance recombinant BMP-2 production in mammalian cell cultures

**DOI:** 10.1186/1753-6561-5-S8-P96

**Published:** 2011-11-22

**Authors:** Aileen J Zhou, Cameron ML Clokie, Sean AF Peel

**Affiliations:** 1 Department of Oral and Maxillofacial Surgery, Faculty of Dentistry, University of Toronto, Toronto, Ontario Canada, M5G 1G6; 2 Induce Biologics Inc, Toronto, Ontario, Canada, M5R 3N8

## Background

Due to their osteoinductive properties, recombinant human bone morphogenetic proteins (rhBMPs) have been used successfully for bone regeneration and replacement. However, the yields rhBMPs yields in mammalian expression systems are very low, resulting in their high cost. BMPs are synthesized as a precursor, proBMP, which undergoes enzymatic cleavage by proprotein convertases (PCs) to form the mature BMP [1]. Furin, an enzyme of the PC family, has shown to cleave BMP-4 [2] and BMP-2 (Zhou et al., unpublished data). This study investigated the effect and mechanism of action of polyarginine furin inhibitor, IND-1, on rhBMP-2 production in mammalian cell lines overexpressing rhBMP-2.

## Materials and methods

Two stable cell lines expressing the *hBMP2* gene, CHO-BMP2 and HEK-BMP2, were cultured in the presence of IND-1 in short-term (24 h, multi-well) and long-term (two-month, perfusion flasks) cultures. The rhBMP-2 produced was characterized by Western blot and its activity assessed using the C2C12 cell-based assay. The amount of proBMP-2 and mature BMP-2 produced was quantified by ELISA. The mRNA level of BMP-2 and furin in cells treated with or without IND-1 was compared by real-time RT-PCR. Cellular uptake of IND-1 was estimated by measuring the fluorescence of cell lysates following incubation with FITC labeled IND-1. Cellular PC activity post IND-1 incubation was measured using the Boc-RVRR-AMC substrate. Furin-specific siRNA was used to knock down the furin expression in CHO-BMP2 cells and its effect on the rhBMP-2 production was determined.

## Results

**Figure 1:**
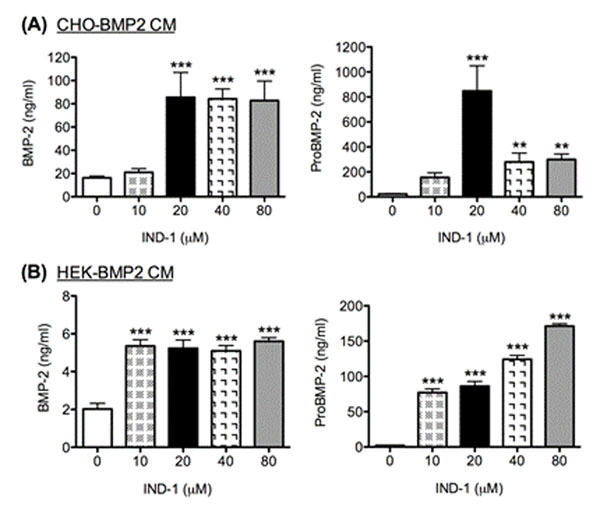
After 24 h incubation with or without IND-1, the amount of proBMP-2 and mature BMP-2 proteins in (A) CHO and (B) HEK conditioned media were measured by proBMP-2 and BMP-2 ELISA. Data are presented as mean ± SEM (*n* = 3 experiments) (**P <*0.01*, ***P <*0.001*).

Stably transfected CHO-BMP2 cells secreted 36 kDa rhBMP-2 dimers that were biologically active. In 24 h cell cultures, IND-1 treated cells produced significantly greater amounts of proBMP-2 (≥ 10-fold, *P* <*0.001*) and mature BMP-2 (≥ 3-fold, *P* <*0.001*) in their conditioned medium (Figure 1). In long-term CHO-BMP2 culture, IND-1 continued to increase the yields of BMP-2 (≥ 50%) and proBMP-2 (≥ 2-fold) without affecting cell growth or viability.

IND-1 treatment had no effect on the mRNA level of BMP-2 and furin, indicating IND-1 affects rhBMP-2 yield post-transcriptionally. While IND-1 was taken up by the cells and inhibited PC activity when added directly to the cell lysates, cells cultured with IND-1 showed no changes in their PC activity at doses 50 times higher than required to affect BMP-2 yields. Furthermore, knockdown of furin at both the mRNA (≥ 80%, *P* <*0.001*) and the protein level (≥ 70%, *P* <*0.001*), did not affect rhBMP-2 yields. These results suggest that furin inhibition is most likely not the mechanism by which IND-1 enhances rhBMP-2 yields.

## Conclusions

The addition of a novel peptide IND-1 to the cell culture medium significantly enhanced the yields of both pro- and mature BMP-2 in stably transfected CHO and HEK cell lines. These increases were sustainable over an extended time period with regular IND-1 treatments. However, the enhanced rhBMP-2 yield is unlikely due to the well-established role of polyarginines as furin inhibitors.
